# Report of a Case

**Published:** 1874-07

**Authors:** Thos. S. Mitchell

**Affiliations:** Georgia


### REPORT OF A CASE.

BY THOS. S. MITCHELL, M.D., OF GEORGIA.

Believing it to be the imperative duty of every regular medical man to give publication to all cases of interest to the profession, therefore, with your permission, I propose to give to my medical brethren a statement of a case whose parallel I have no knowledge of, nor my medical friends who kindly assisted me in the case. I only propose to give a general history of the case, and the result, and let my medical brethren say what the case was, and what killed the boy. To come direct to my case, I propose to commence at the beginning and go to the "finale."

About eighteen months ago I was called in haste to visit Wm. Harris, colored, age twenty-one years, who had "cramp colic." I found him almost in complete tetauus from very excruciating and intense pain; I found upon examination the right testicle about three-fourths of an inch to the left of the umbilicus—presenting *immediately under the skin.* I gave him twenty grains chloral hydrat, at one dose; used a liniment and formentations; followed by a brisk cathartic.

Called again the next morning; found the medicine had acted well, and the boy "well;" no trace of the nut to be found, it had disappeared entirely from view and feeling. He went to work the next day and continued well and exceedingly stout until last Friday, April 3d, 1874.

About twelve years ago the boy and others were playing, "*pushing our nuts up in our bellies and I beat them all.* Mine never come back any more in my bag." This boy has been exercising very violently on the horizontal bar, turning as many as seventeen back somersaults at a time on last Sunday.

On last Friday noon I was called again to visit "Billie." I found him suffering as before; the pain equally as intense as before; I prescribed the same remedies as before; gave him ease but no action from the bowels, save a small quantity of fæces from the rectum—brought off by enemas. I was completely "trumped." I invited my friends Drs. W. P. McGehee and John W. Cameron to visit the case, they both thought the testicle could be replaced by taxis. I told them I had failed and would like for them to do it. After a fair trial both failed to get it back even in its "old bed," much less into the scrotum. Dr. McGehee advocated the knife as the only resort. Dr. Cameron thought with me, to wait a little longer and trust to nature. If that failed to use chloroform and replace it by taxis. I waited six hours and was summoned again in haste. "Billie was dying." I found him threatened with lock-jaw. I gave him chloroform, "full" to complete stupor, and then did everything I could to relieve the boy. I failed again. I left him sleeping with anodynes for the night.

Sunday morning I found him no better; was stupid from anodyne; no nourishment taken since Friday morning—breakfast; condition unchanged, save the distention of the abdomen, which gradually pushed the nut over to the right side in the right illiac region, where it remained until death.

On Monday morning I summoned to my assistance the following medical gentlemen: Drs. W. W. Bruce, J. S. McCants and J. W. Cameron. All with one accord agreed in every particular as to the operation, and not thinking of any opposition, I was busy preparing my instruments, etc., for the operation, when to our great and complete astonishment, the family refused their consent to the operation, and no amount of reasoning, arguments, or outside influence would avail anything. We were as a body threatened by the negroes to mob us, to prosecute us, or assassinate us if we operated against their wishes. We all retired from the case in shame and disgust from their ignorance and superstition. We left the boy to die from the ignorance of his friends, he lived on until Thursday, one o'clock; and died in great agony. Dr. C. visited him with me until he died. We refused to offer any further treatment after we retired from the case, but visited him from pure curiosity. Dr. T. F. Brewster came to town during this time; I invited him to see the boy with me as it was a very extra ordinary case. He coincided with my other friends that the knife was the only hope for the boy's life.

On Monday night an old retired practitioner was called to the case. He sent for me to meet him in consultation: this of course I refused to do. He took charge of the case with some promises to cure. He commenced by the application of a blister and the internal administration of quinine and his "long green," a secret remedy he claims to be good for all the "ills flesh is heir to," but "long green" failed as all men of common knowledge of the case knew it would. The boy died in great agony, calling for me to help him to the hour of his death. "Billie is dead." My friends, many of them the first citizens in the county used all their influence, in every way; offered considerable sums in money to get an autopsy, but ignorance, superstition and meanness prevail over reason and common sense. "Billie" is buried among the everlasting mysteries of the dead.

In conclusion I desire to ask my medical brethren, what (aside from palpable wrong, meanness and ignorance of his friends, "so-called" and relations,) killed my patient? was it an invaginated bowel, or was it strangulated hernia, produced by the bowels trying to follow the testicle out through the abdominal muscles, forming a knuckle in the bowels, thereby destroying the patient by acute peritonitis, etc.? Will some one give us more light on this case?

---

A New Destroyer of the Hair.—Under the above title, Dr. Boettger, in the *Memorabilien*, says that we possess a new material for destruction of hair, of a most suitable description, in a mixture of one part of crystallized sulphydrate of sodium with three parts of fine carbonate of lime mixed and reduced to a very fine powder. This mixture may be kept any length of time without alteration in well-closed bottles. When moistened with a drop of water and laid by means of the back of a knife on the part of the skin covered with hair, we in a few minutes find the thickest hair turned into a soft mass, easily removed by means of water. If it remains on the part long it will cause a slight irritation of the skin.—*London Medical Record.*

Case of Neuralgia of the Testes Cured by Electricity.—A young man free from all syphilitic disease experienced such intense pain in the testes that he urgently asked Dr. Felippi to perform castration. The case was carefully made out to be neuralgia, independent of any affection of the testicle or of any accumulation of fecal matter, and in five sittings the patient was entirely cured. Dr. Felippi made use of a weak and direct constant current. —*L'Imparzial.*

